# Cooperative nutrient accumulation sustains growth of mammalian cells

**DOI:** 10.1038/srep17401

**Published:** 2015-12-01

**Authors:** Sungmin Son, Mark M. Stevens, Hui Xiao Chao, Carson Thoreen, Aaron M. Hosios, Lawrence D. Schweitzer, Yaochung Weng, Kris Wood, David Sabatini, Matthew G. Vander Heiden, Scott Manalis

**Affiliations:** 1 Koch Institute for Integrative Cancer Research, Massachusetts Institute of Technology, Cambridge, MA.; 2 Department of Biology, Massachusetts Institute of Technology, Cambridge, MA.; 3 Department of Mechanical Engineering, Massachusetts Institute of Technology, Cambridge, MA.; 4 Department of Biological Engineering, Massachusetts Institute of Technology, Cambridge, MA.; 5 Computational and Systems Biology Initiative, Massachusetts Institute of Technology, Cambridge, MA; 6 Whitehead Institute for Biomedical Research Nine Cambridge Center, Cambridge, MA; 7 Dana-Farber Cancer Institute, Boston, MA.

## Abstract

The coordination of metabolic processes to allow increased nutrient uptake and utilization for macromolecular synthesis is central for cell growth. Although studies of bulk cell populations have revealed important metabolic and signaling requirements that impact cell growth on long time scales, whether the same regulation influences short-term cell growth remains an open question. Here we investigate cell growth by monitoring mass accumulation of mammalian cells while rapidly depleting particular nutrients. Within minutes following the depletion of glucose or glutamine, we observe a growth reduction that is larger than the mass accumulation rate of the nutrient. This indicates that if one particular nutrient is depleted, the cell rapidly adjusts the amount that other nutrients are accumulated, which is consistent with cooperative nutrient accumulation. Population measurements of nutrient sensing pathways involving mTOR, AKT, ERK, PKA, MST1, or AMPK, or pro-survival pathways involving autophagy suggest that they do not mediate this growth reduction. Furthermore, the protein synthesis rate does not change proportionally to the mass accumulation rate over these time scales, suggesting that intracellular metabolic pools buffer the growth response. Our findings demonstrate that cell growth can be regulated over much shorter time scales than previously appreciated.

The coordination of metabolic processes to allow increased nutrient uptake and utilization for macromolecular synthesis is central for cell growth. Although studies of bulk cell populations have revealed important metabolic and signaling requirements that impact cell growth on long time scales, whether the same regulation influences short-term cell growth remains an open question^1^,^2^. The dynamics of cell growth – accumulation of cell mass – are largely unexplored because it has not been possible to directly measure growth over time scales that are small compared to the interdivision time. Here, we investigate cell growth by monitoring how the mass of single suspension cells respond to nutrient depletion over minute time scales. For these studies, we take advantage of the suspended microchannel resonator (SMR) to precisely determine single-cell buoyant mass accumulation rate within 20 minutes^3^. By rapidly exchanging the media surrounding a cell, we can monitor the change in buoyant mass accumulation rate that results from depletion of a particular nutrient. By correlating these findings to population measurements of protein synthesis and cell signaling we show that cells can instantaneously alter growth rates upon nutrient depletion in a manner that is independent of the mechanisms described to control growth over longer time scales.

Buoyant mass accumulation reflects any change of total cell contents caused by molecules being exchanged with the extracellular environment (Fig. 1a). This is a meaningful representation of cell growth for several reasons. First, metabolites and macromolecules such as nucleic acids, proteins, and lipids, rather than ions or water, are the primary contributors to cellular buoyant mass because they are far more concentrated in cells than in surrounding fluid. Second, buoyant mass represents the summation of all molecular contents of a cell, thereby avoiding possible biasing in growth measurements that use particular molecular content, such as protein, as a proxy for the total molecular contents^4^. Third, a change in buoyant mass reflects the net flux of molecules across the cell membrane regardless of the type of flux–diffusion, active transport, or endo-/exo-cytosis. Combining this knowledge with the SMR’s precision to measure buoyant mass within 0.05% error (Supplementary Fig. 1) enables the direct measurement of single-cell mass accumulation rate (MAR) over a period of 20 minutes.

## Results

### Reduction of mass accumulation rate following nutrient depletion

**Figure 1:**
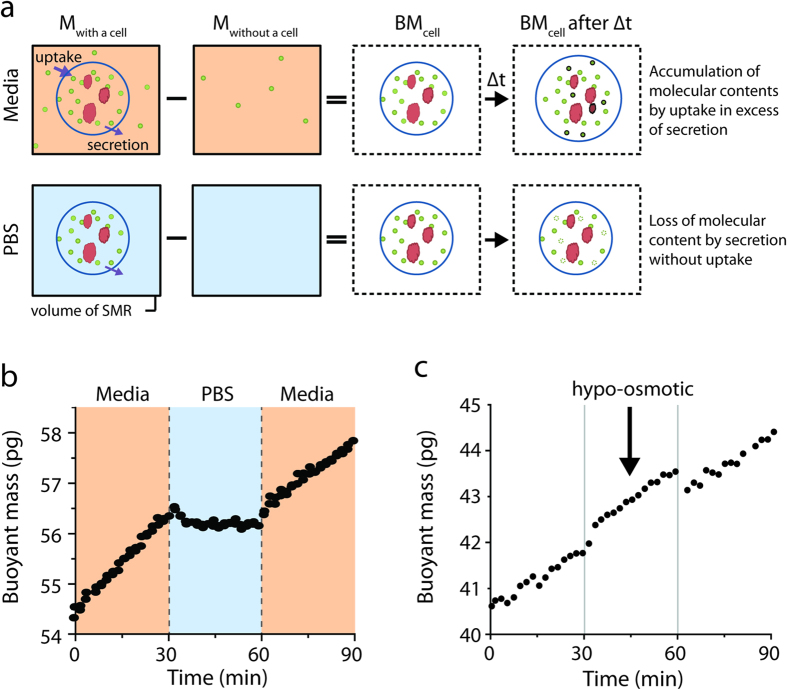
The SMR measures instantaneous accumulation of molecular contents in a single cell. (**a**) The difference in SMR mass (black box) with or without a cell is the buoyant mass of a cell. Since ion concentration inside and outside the cell is approximately the same, the buoyant mass of a cell is determined by molecular contents such as small molecules (green circle–metabolites or amino-acids) or macromolecules (red globule–proteins, lipids, or nucleic-acids). In media, cellular uptake exceeds secretion thus molecular contents accumulate over time (dark green and red indicate newly acquired molecules). In PBS when all environmental nutrients are absent, cells only secrete molecules and molecular contents decrease (dim green indicates the lost molecules). (**b**) A cell’s buoyant mass is measured while its surrounding is rapidly switched from media to PBS (t = 30 minutes) and back to media (t = 60 minutes). The orange and blue background mark culture media and PBS, respectively, as in (**a**). The transient buoyant mass change at the transition results from the density and osmolarity mismatch between the media and PBS. The responses from additional cells are shown in Supplementary Fig. 3. (**c**) Changing the density and osmolarity can offset the cell’s buoyant mass but the mass accumulation rate remains unchanged. Hypo-osmotic condition was achieved by diluting standard RPMI media to 20 mM lower osmolarity with deionized water.

We utilized cells from one of three suspension cell lines that are amenable for these measurements: L1210 murine lymphocytic leukemia cells, FL5.12 murine pro-B-cell, and Jurkat human T-lymphocyte cells, all of which have been previously investigated in studies related to cell cycle^5^,^6^, metabolism^1^,^7^,^8^, and T cell signaling^9^, respectively. Although there are differences between the bulk culture and SMR environments (e.g. aeration and nutrient sharing between cells), cell growth in the SMR system is similar to what is observed in bulk culture in terms of size, inter-division time, and mass accumulation rate^3^. To determine whether we could precisely measure MAR while modifying nutrient availability within seconds (Supplementary Fig. 2), we exchanged the media of growing FL5.12 cells for phosphate buffered saline (PBS), thereby removing all nutrients (Fig. 1b). Cells that grew at rates typical for these cells prior to depletion acquired a negative MAR in less than two minutes (Supplementary Fig. 3 and 4), consistent with the expectation that some nutrient input is required for mass accumulation. These findings are also consistent with continued metabolism of existing cell material to sustain survival in the absence of nutrient uptake. Importantly, growth could be restored by exchanging the cell back into normal nutrient-containing media suggesting that viability is not compromised even when all nutrients are removed over these time scales. Because depleting small-molecule metabolites can alter the osmotic pressure, there was the potential that osmotic pressure change could influence MAR. To test this possibility, we measured growth of cells for 30 minutes in standard media, followed by 30 minutes in media where total osmolarity was reduced by 20 mM and found that MAR remained constant while in hypo-osmotic conditions (Fig. 1c), arguing that changes in MAR observed in PBS were the result of nutrient depletion.

We next determined the relationship between MAR and long-term proliferation by depleting materials in culture media previously shown to impact long-term proliferation. Macromolecules in fetal bovine serum (FBS), like cytokines and growth factors, may, in some cell types, contribute directly to cell mass accumulation through endocytic pathways in addition to their well established role in growth signaling^10^. As expected, depletion of FBS in bulk culture induced a long-term proliferation arrest in both FL5.12 and L1210 cells (Supplementary Fig. 5d). Surprisingly, depletion of FBS did not change the MAR in either cell type (Supplementary Fig. 6). Thus, despite the critical roles of growth factors and other macromolecules for proliferation over long time scales, the availability of small molecule nutrients alone is sufficient to maintain cell growth over short periods.

**Figure 2:**
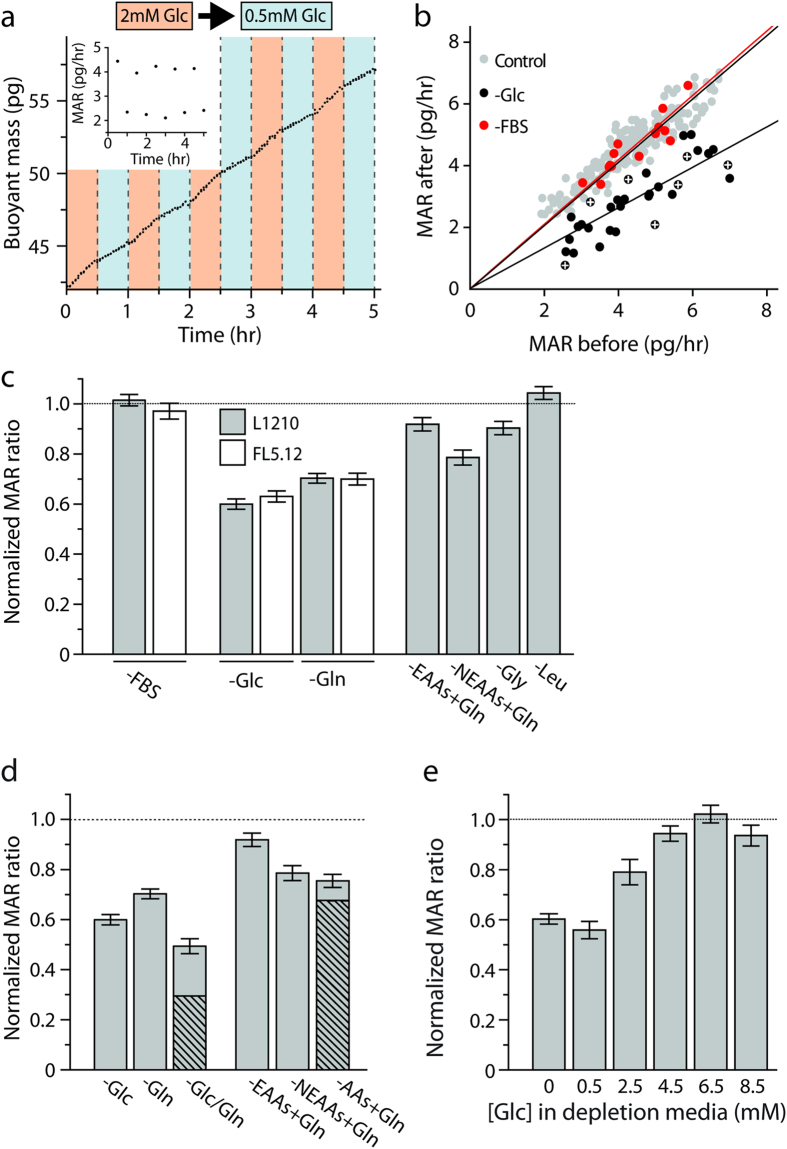
Instantaneous change of mass accumulation rate (MAR) upon nutrient depletion. (**a**) Buoyant mass accumulation of a FL5.12 cell while the environmental glucose concentration changes between 2 mM and 0.5 mM every 30 minutes. *Inset:* mass accumulation in each period is linearly fit to determine the MAR. (**b**) For each cell, MAR is determined over a 30-minute period in normal media and again for the same period in depleted media. MAR change is determined by fitting a linear line with zero-intercept (black or red line). White crosses represent the MAR measurement error. For control, MARs are determined for a cell grown only in normal media (grey circle). (**c**) Normalized change of MAR for various nutrient depletions. Each column indicates the slope of a linear fit to the single-cell growth rate ratios as shown in (**b**) (see Supplementary Fig. 6 for the single cell data). (**d**) MAR reduction is not additive when both glucose and glutamine are depleted. Grey columns indicate the reduction of MAR upon depletion of various nutrients as measured by the SMR. Dashed columns indicate the expected reduction of MAR if the responses were additive. (**e**) Normalized change in MAR shows that mass accumulation reduction occurs even in incomplete depletions and is dose-dependent. All error bars indicate the standard error of the linear fit estimation.

Glucose (Glc) and glutamine (Gln) are key anabolic and energetic substrates essential for long-term proliferation^11^. We confirmed that over the course of several hours, depleting either substrate reduces the proliferation of both L1210 and FL5.12 cells (Supplementary Fig. 5). However, in contrast to serum depletion, SMR measurements revealed a rapid, larger than expected, and reversible reduction in MAR (37–52% and 30–34% for Glc and Gln, respectively) that is proportional to the growth rate prior to depletion (Fig. 2a–c, Supplementary Fig. 6 and 7). Depletion of non-essential amino acids (NEAAs) induced a nearly 2-fold larger reduction in MAR when compared to essential amino acids (EAAs), despite the fact that cell proliferation on long time scales is more sensitive to essential amino acid availability (Fig. 2c, Supplementary Fig. 5 and 6). Depletion of a single non-essential amino acid, glycine (Gly), whose consumption was recently found to correlate with cancer cell proliferation^12^, induced a reduction in MAR that was equal to half the effect measured when all non-essential amino acids were depleted. Finally, depletion of leucine (Leu), which is known to inhibit mTORC1 signaling within one minute^13^, did not induce any change in mass accumulation. This suggests that short-term changes in growth may reveal different aspects of metabolic growth requirements than can be discerned by long-term (hours to days) changes in proliferation.

### Depleting a particular nutrient alters the uptake of other nutrients

We wondered if the reduction in MAR following specific nutrient depletion resulted solely from the reduction in uptake of that particular nutrient, thereby only reflecting the accumulation rate of that nutrient in an unperturbed state. To test this, we performed simultaneous depletion of both glucose and glutamine on L1210 cells and found a 50% reduction in MAR relative to the initial rate. This value is less than an additive reduction in growth rate, which we would predict to be ~70% based on the reduction in MAR observed when each nutrient is depleted individually (Fig. 2d, Supplementary Fig. 6). When other nutrients pairs were co-depleted, such as essential and non-essential amino acids, the reduction in MAR was also not additive from what is observed with separate nutrient depletions. This lack of an additive reduction in MAR suggests that the decreased growth of cells following nutrient depletion is more complex than losing the contribution of a particular nutrient to new cell mass. In order to place an upper limit on the rate of mass accumulation of these metabolites we next measured the rate of ^14^C-labeled glucose and glutamine incorporation into macromolecules in bulk culture and found that the accumulation rates for glucose and glutamine each account for less than 17% of the total accumulation rate (Supplementary Fig. 8). In terms of nutrient uptake rates, a cell in bulk culture and an isolated cell in the SMR are likely to share the same upper limit because the size and inter-division time are the same in both conditions^3^. Thus, accumulation of a specific nutrient alone cannot explain the >30% reduction in MAR following glucose or glutamine depletion. Taken together, these findings suggest that the larger than expected reduction in MAR following individual nutrient depletion is the result of concomitant reduction of mass accumulation from other sources. This indicates that, for the cells tested here, accumulation of nutrients is cooperative for cell growth. Notably, the reduction in MAR occurred even in the absence of complete depletion, as similar results were obtained when glucose concentration fell below a specific level in the physiological range (Fig. 2e).

### Canonical activity of nutrient sensing pathways are not activated upon reduction of mass accumulation rate

**Figure 3:**
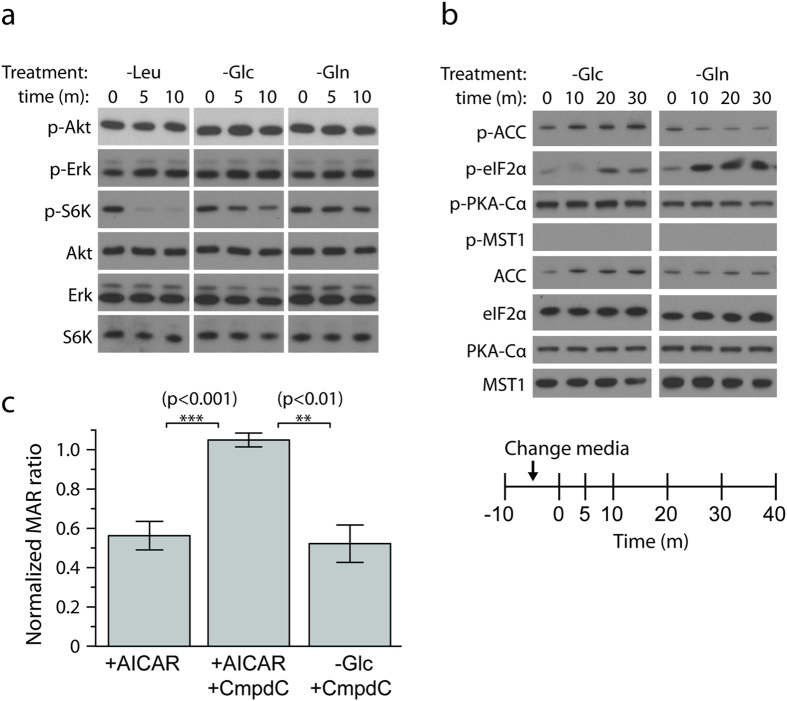
The known growth and nutrient sensing pathways are not involved in the instantaneous growth response. (**a**) Immunoblot of Akt (Ser473), Erk (Thr202/Tyr204), and S6K (Thr389) in L1210 cells within 10 minutes after the depletion of leucine, glutamine, or glucose. (**b**) Immunoblot of phosphorylation of the downstream target of AMPK, ACC (Ser79), as well as eiF2α (Ser51), PKA (Thr197), and MST1 (Thr183) in L1210 cells within 30 minutes after the depletion of glucose or glutamine. The schematic shows the time of depletion and subsequent fixation of cells. (**c**) AMPK is not necessary for the growth response upon glucose depletion. AICAR induces MAR reduction through phosphorylation of AMPK, Compound-C successfully inhibits AMPK activity as indicated by the abolishment of MAR reduction, but Compound-C does not abolish MAR reduction in the case of glucose depletion. p-values were calculated by using Welch’s t-test.

In order to determine if cooperative mass accumulation is mediated by established growth and nutrient sensing pathways, we used immunoblotting with phospho-specific antibodies to monitor acute changes in activity of the mTORC1, AMPK, ERK, AKT, PKA, MST1, and eiF2α pathways upon depletion of glucose, glutamine, or leucine (Fig. 3a,b, Supplementary Fig. 9). We also assessed whether autophagy or apoptosis is induced based on the immunoblotting of LC3 and caspase-3 cleavage, respectively. Depletion of glucose or glutamine did not induce any LC3 dimerization or caspase-3 cleavage over a 12-hour interval, suggesting that these cellular programs are not involved in the observed MAR responses (Supplementary Fig. 10). mTORC1 activity was assessed by probing S6K1 phosphorylation, an immediate downstream target that is rapidly inhibited upon leucine depletion but not upon glucose or glutamine depletion. The observation that MAR decreased most significantly upon depletion of glucose, but did not decrease upon leucine depletion (Fig. 2c) suggests that the MAR responses are not mediated by mTORC1-dependent regulation. AKT, ERK and PKA, well establish regulators of growth^14^, showed no change to their phosphorylation state following glucose or glutamine depletion on relevant timescales, arguing against the involvement of these signaling pathways in mediating the short term growth response to nutrient depletion (Fig. 3a,b, Supplementary Fig. 9). Immunoblots of the mammalian HIPPO homolog MST1 also did not show changes in phosphorylation over relevant timescales (Fig. 3b, Supplementary Fig. 9). However, these findings must be interpreted cautiously as it is possible that small contributions from multiple nutrient sensing pathways, unresolvable by western blots, could mediate the reduction in mass accumulation rate.

AMPK activation was observed within 10 minutes of glucose depletion in both FL5.12 and L1210 cells (Fig. 3b, Supplementary Fig. 9a), a finding consistent with glucose supporting ATP production. We independently tested if AMPK is responsible for changes in MAR (Fig. 3c, Supplementary Fig. 11). When AMPK activity was induced with the AMPK activator AICAR (5-aminoimidazole-4-carboxan ribonucleotide)^15^, cells change MAR, suggesting that AMPK activation might contribute to the decrease in MAR upon nutrient depletion. To test this possibility further, we checked whether inhibition of AMPK by compound-C^16^ abolishes growth reduction upon glucose depletion. Despite any AMPK-independent inhibitory effect of compound-C on cell metabolism^17^, compound-C does not alter MAR on its own yet it abolishes MAR reduction induced by AMPK activation. Interestingly, cells decrease their MAR upon glucose depletion even when AMPK phosphorylation is inhibited by compound-C, indicating that AMPK is not required to mediate the change in MAR following glucose depletion. Taken together, these data suggest that classical nutrient-sensing pathways are not required to control cell growth rate over short time scales.

Alternatively, the growth response could be regulated by a membrane-level nutrient sensor such as those described in yeast^18^. When we supplemented 2-deoxy-glucose (2DG) upon depletion of glucose, however, cells still significantly decrease their mass accumulation rate (Supplementary Fig. 12), suggesting the mechanism of sensing decreased glucose metabolism is downstream of glucose-6-phosphate.

### Reducing protein synthesis rate does not necessarily reduce mass accumulation rate

**Figure 4:**
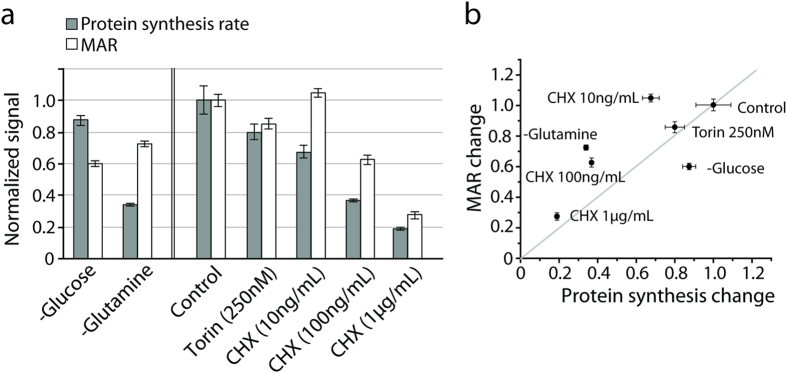
Growth can be temporarily decoupled from protein synthesis upon nutrient depletion. (**a**) Grey columns indicate normalized protein synthesis rate measured by radiolabel incorporation. White columns indicate normalized MAR measured by the SMR. Following glucose or glutamine depletions, both protein synthesis and MAR were measured for 30 minutes immediately after depletion. Following drug treatment, protein synthesis and MAR were measured 2 hours after inoculation. MARs were determined over a 15-minute interval in each condition. The mean MAR was normalized by the mean MAR of untreated cells. Number of cells measured in the SMR is 84 (control), 68 (Torin 250 nM), 67 (CHX 10 ng/ml), 89 (CHX 100 ng/ml), 53 (CHX 1ug/ml). Error bars indicate the variation of mean from triplicate measurement (protein synthesis) or standard error (mass accumulation rate). (**b**) The results in (**a**) are plotted to show the relationship between reduction in MAR and protein synthesis.

We found that glutamine depletion weakly induces phosphorylation of eiF2α over relevant timescales (Fig. 3b). eiF2α phosphorylation is known to repress protein synthesis in response to nutrient stresses including amino acid deprivation^19^. To determine if eiF2α is coupled to the observed reduction in MAR through repression of protein synthesis, we first tested if the protein synthesis rate also reduces instantaneously after glutamine depletion (Fig. 4). In fact, within 30 minutes following glutamine depletion, protein synthesis was decreased by 66.0% compared to only 12.6% upon glucose depletion. Next, we tested if decreased protein synthesis alone was sufficient to reduce MAR. Specifically, we tested if either targeted inhibition of mRNA translation using cycloheximide (CHX), or inhibition of mTORC1 signaling to reduce protein synthesis has an effect on MAR. In both cases, reduced protein synthesis has some effect on MAR, but contrary to our expectations that cell growth and protein synthesis would be tightly coupled^20^, changes in protein synthesis do not correlate with changes in MAR (Fig. 4). When translation was inhibited directly with CHX, MAR dropped proportionally to, but less than, the reduction in protein synthesis. In contrast, when mTORC1 was inhibited, both protein synthesis and MAR decrease in equal proportion. Therefore, changes in protein synthesis can induce changes in MAR, but cell growth and protein synthesis can be decoupled. Notably, while a larger drop in protein synthesis relative to the drop in MAR was also observed following glutamine depletion, the converse was true in the case of glucose depletion, demonstrating further that a reduction of MAR does not require a proportional reduction in the rate of protein synthesis.

## Discussion

Although most cells within multicellular organisms are thought to experience relatively constant nutrient levels, some cells likely experience rapid fluctuations in nutrient availability and metabolic flexibility is crucial for ensuring both cell growth and survival in these contexts^2^. For example, immune cells must rapidly migrate in or out of circulation to different environmental niches. Cooperative nutrient accumulation and the decoupling of cell growth from the production of specific macromolecules such as proteins, demonstrated to act on minute timescales in the cells tested, could provide an efficient way to ensure that growth metabolism is only engaged when there are adequate nutrients to effectively increase cell mass. For instance, because the pools of important metabolic cofactors such as NAD(P)(H) are small relative to flux through anabolic pathways, they must be constantly regenerated to support various processes. Thus, if one nutrient is critical to support NAD(P)(H) production in order to utilize a second nutrient to produce cell mass, nutrient depletion could have a larger effect on mass accumulation than simply removing that nutrient’s contribution to new cell mass. The fact that growth often recovers immediately after nutrient repletion implies that cell growth is robust even when the primary nutrients are temporarily depleted. Furthermore, consistent with some nutrients playing a less important role in cell growth, we find that the short-term growth response is highly nutrient specific with depletion of some fuels having minimal effects on MAR.

## Methods

### Cell culture

L1210 and FL5.12 cells were maintained in RPMI media (Invitrogen, Cat#11875–119) supplemented with 10% dialyzed FBS (Tissue Culture Biologicals, Cat#101DI, Lot#109615). For the depletion of glucose, glutamine, or both, RPMI lacking each metabolite or both was purchased (Invitrogen, Cat#11879–020, 21870–076, Biological Industries, Cat#01–101–1A). For all other depletions, a custom base media lacking all amino acids, pyruvate, and glucose was used (Cellgro, Project#11–PB–090A), and the desired media was constituted by adding back the necessary metabolites individually. To deplete a particular metabolite from bulk cell culture, cells were washed three times in PBS and resuspended in the media lacking the metabolite. To prevent the addition of unspecified amount of metabolites in FBS, the culture and depletion media were supplemented with dialyzed FBS. To monitor cell volume and proliferation in depleted culture, cells were measured using Coulter Counter following depletion (Supplementary Fig. 5). For the single-cell depletion experiments in the SMR, all depleted media was supplemented with mannitol (Sigma-Aldrich, Cat#M4125) at the concentration matching to the original concentration of the depleted metabolites for osmotic compensation.

### Suspended microchannel resonator (SMR) and high-precision measurement of mass accumulation rate (MAR)

The principles behind the SMR have been previously described^3^,^21^. Briefly, cells in suspension are transported through the SMR and the resulting frequency shift is proportional to cell buoyant mass. The SMR can resolve the instantaneous rate of mass accumulation for a single cell in 20 minutes within an error of 0.5% provided that the cell is weighed every two minutes (Supplementary Fig. 1). To calculate the MAR from measurement of frequency shift versus time, the raw data was calibrated by polystyrene beads with a known mass (Bangs Laboratories, Cat#NT21N, NT25N) and linearly fitted.

### Protein synthesis rate measurement by ^35^S incorporation

Cells were seeded into 12-well plates at 300 k/mL and allowed to culture overnight in standard growth conditions. For depletion measurements, cells were spun down at 1200 g for 3 minutes, washed twice in 37 **°**C and resuspended in osmotically balanced RPMI media lacking the metabolite of interest/cysteine/methionine. For protein synthesis inhibition measurements, cells were treated with the appropriate concentration of compounds 2 hour prior and washed twice with cysteine/methionine-free RPMI (Sigma Aldrich, Cat#R7513). In both measurements, cells were resuspended in 1 mL of the same media that they were washed in and supplemented with 110 μCi (10 μL, 11 μCi·μL^−1^) of EasyTag EXPRESS ^35^S protein labeling mix (Perkin Elmer, Cat#NEG772007MC). Cells were incubated with radiolabel for the next 30 minutes at 37 **°**C, washed twice at 1200 g with 10 mL ice-cold PBS, lysed in 150 μL RIPA buffer and spun at 13000 g to isolate soluble fractions. Lysates were spotted on Whatman paper, precipitated with 5% trichloroacetic acid to extract protein, washed twice for 5 minutes with 10% trichloroacetic acid, washed twice for 2 minutes with ethanol, washed once for 2 minutes with acetone and dried at room temperature. All lysate manipulation was carried out in ice-cold buffers in a cold room. The total ^35^S incorporation was measured using a Beckman Coulter LS6500 Scintillation Counter. Total protein content of lysates was determined using a Bradford Assay (Bio-Rad).

### Glucose and glutamine accumulation rate by ^14^C incorporation

Cells were seeded at 250 k/mL into 5 mL cultures with freshly prepared RPMI and allowed to recover for about 2 hours. Next, 4 μCi/mL (final) of [U-^14^C]-glucose (fully labeled) or 0.2 μCi/mL (final) of [U-^14^C]-glutamine (American Radiolabeled Chemicals, Cat#ARC0122G and ARC0196) was added, and the cells were sampled at 60-minute intervals. Samples were washed in ice cold PBS, and total ^14^C incorporation measured using a Beckman Coulter LS6500 Scintillation Counter. Incorporation data were converted to units of pg/hr from the specific activity of the ^14^C-labeled nutrient and its proportion in the media relative to the unlabeled nutrient^22^,^23^.

## Additional Information

**How to cite this article**: Son, S. *et al.* Cooperative nutrient accumulation sustains growth of mammalian cells. *Sci. Rep.* **5**, 17401; doi: 10.1038/srep17401 (2015).

## Supplementary Material

Supplementary Figures
