# Author Correction: Cooperative nutrient accumulation sustains growth of mammalian cells

**DOI:** 10.1038/s41598-020-69376-2

**Published:** 2020-07-21

**Authors:** Sungmin Son, Mark M. Stevens, Hui Xiao Chao, Carson Thoreen, Aaron M. Hosios, Lawrence D. Schweitzer, Yaochung Weng, Kris Wood, David Sabatini, Matthew G. Vander Heiden, Scott Manalis

**Affiliations:** 1 Koch Institute for Integrative Cancer Research, Massachusetts Institute of Technology, Cambridge, MA USA; 2 Department of Biology, Massachusetts Institute of Technology, Cambridge, MA USA; 3 Department of Mechanical Engineering, Massachusetts Institute of Technology, Cambridge, MA USA; 4 Department of Biological Engineering, Massachusetts Institute of Technology, Cambridge, MA USA; 5 Computational and Systems Biology Initiative, Massachusetts Institute of Technology, Cambridge, MA USA; 6 Whitehead Institute for Biomedical Research Nine Cambridge Center, Cambridge, MA USA; 7 Dana-Farber Cancer Institute, Boston, MA USA

Correction to: *Scientific Reports* 10.1038/srep17401, published online 01 December 2015.

This Article contains an error. In Supplemental Figure 9A, the immunoblot of P-PKA-Cα following glutamine depletion was inadvertently duplicated from the immunoblot of P-PKA-Cα following glucose depletion. The correct Supplementary Figure 9 appears below as Figure 1.

**Figure 1:**
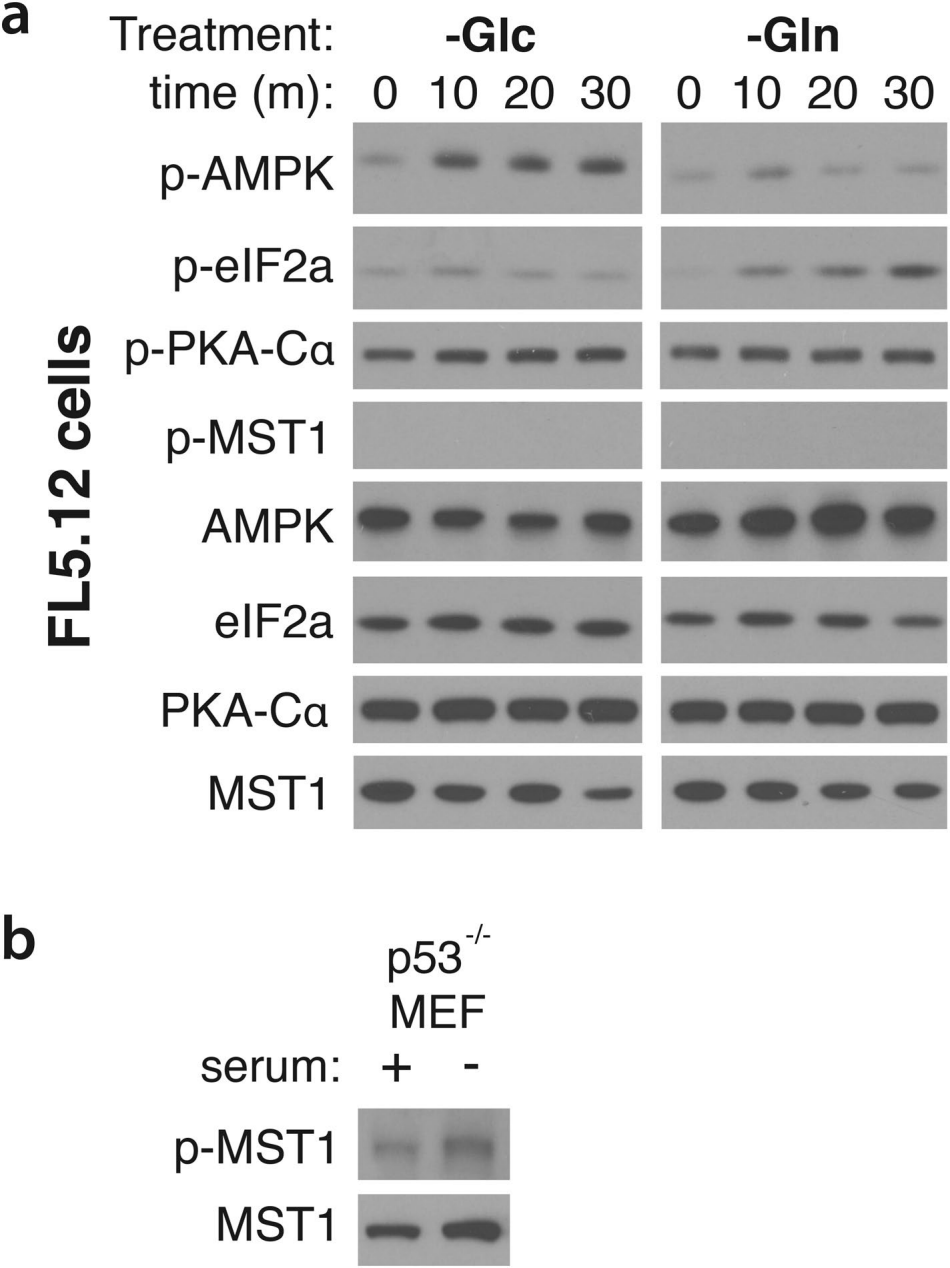
.

The full blots for figures used appear below as Figure 2.

**Figure 2:**
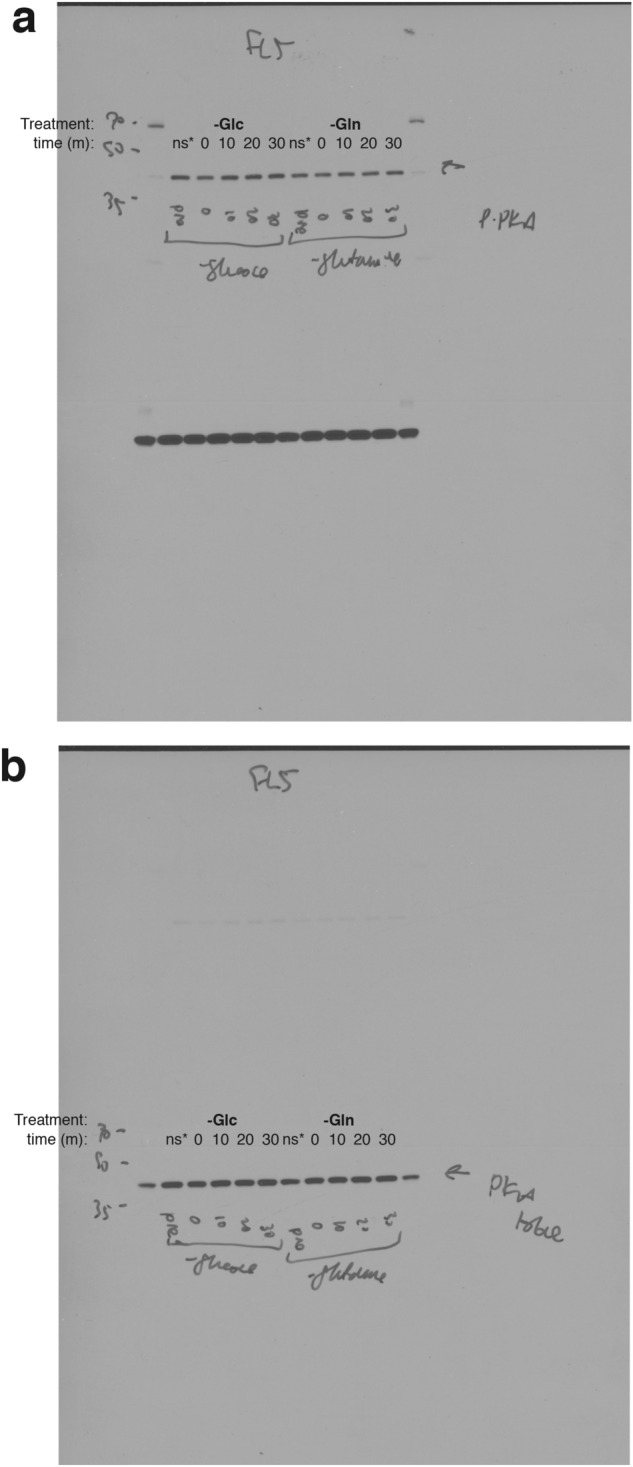
Original scan immunoblots used in Supplemental Fig. 9a. (**a**) exposure of uncropped immunoblots used for p-PKA-Cα. (**b**) exposure of uncropped immunoblots used for total PKA-Cα. ns*: non-stimulated, not used in the final figure.

