# Supplementary material for: Cooperative nutrient accumulation sustains growth of mammalian cells
Source: Sci Rep. 2015 Dec 1;5:17401. doi: 10.1038/srep17401 (PMC4665017; doi:10.1038/srep17401)
Supplement: Supplementary Figures [file srep17401-s1.docx]

Supplementary Information

Cooperative nutrient accumulation sustains growth of mammalian cells

Sungmin Son^1,3*^, Mark M. Stevens^1,2*^, Hui Xiao Chao^1^, Carson Thoreen^2,6^, Aaron M. Hosios^1,2^, Lawrence D. Schweitzer^2,6^, Yaochung Weng^1,5^, Kris Wood^6^, David Sabatini^2,6^, Matthew G. Vander Heiden^1,2,7^, Scott Manalis^1,4,5,†^

^1^Koch Institute for Integrative Cancer Research, ^2^Department of Biology, ^3^Department of Mechanical Engineering, ^4^Department of Biological Engineering, ^5^Computational and Systems Biology Initiative, Massachusetts Institute of Technology, Cambridge, MA, ^6^Whitehead Institute for Biomedical Research Nine Cambridge Center, Cambridge, MA, ^7^Dana-Farber Cancer Institute, Boston, MA

*These authors contributed equally to this manuscript

^†^To whom correspondence should be addressed

Email: srm@mit.edu

**
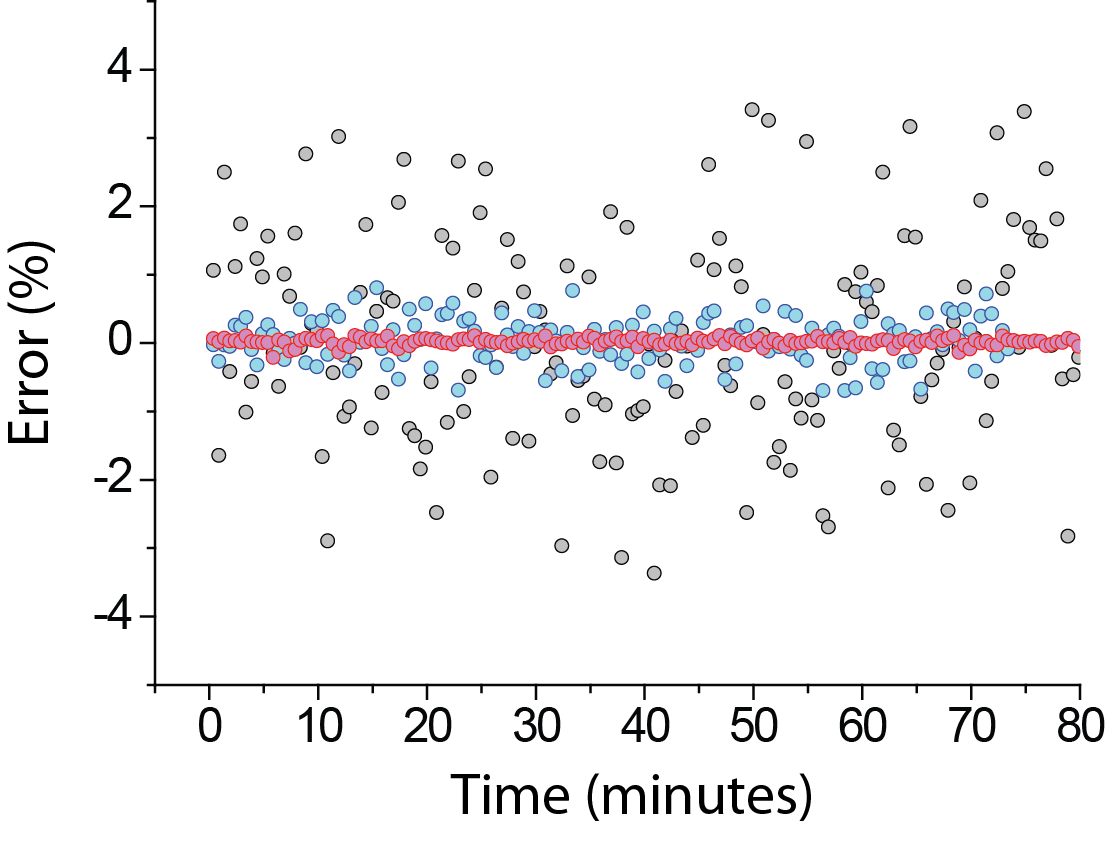
**

**Supplementary Figure 1. SMR measurement error of single-cell buoyant mass**.

Non-growing single L1210 cells are measured in three different SMR operations, sampled at two times per minute: first vibrational mode (grey circles), first vibrational mode combined with a hydrodynamic focusing (blue circles), and second vibrational mode (pink circles). The root-mean-square (RMS) errors of the raw data are 1.51%, 0.34%, and 0.048%, respectively. In the SMR, a cell can wander in a direction orthogonal to the flow stream, creating what is called position-dependent-error that limits the buoyant mass precision to 1.51% without any operational improvement. Previously, the error was reduced to 0.34% by deploying a form of hydrodynamic focusing that confines the flow path of a cell as it travels through the SMR^1^. In the current study, the position-dependent-error is fundamentally removed by operating the SMR in the second vibrational mode and measuring the buoyant mass from the antinode^2^. As a result, the RMS error is reduced to 0.048%.


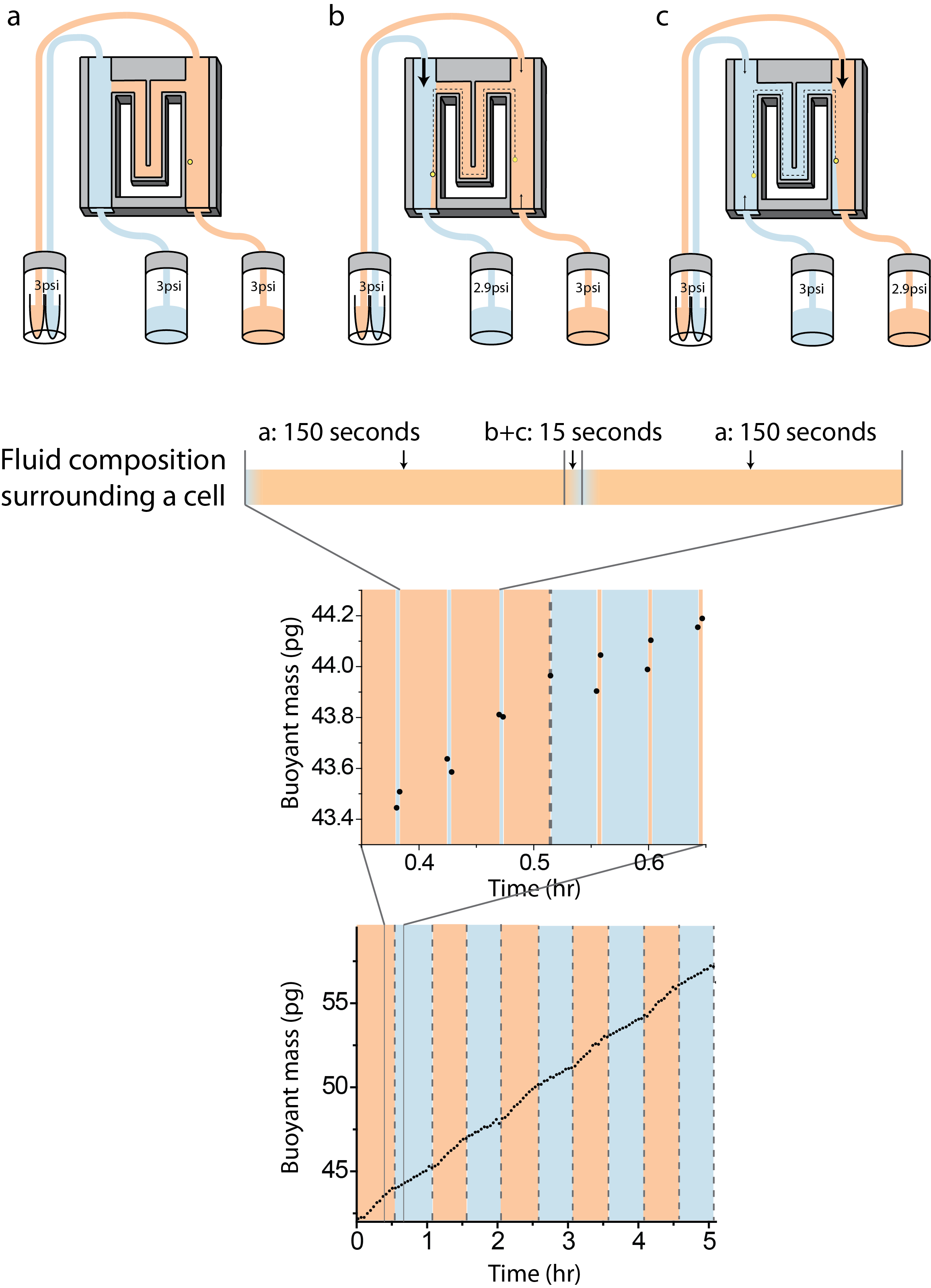


**Supplementary Figure 2. Rapid exchange of the media surrounding a single-cell.**

First, two different media were filled in two large bypass channels (nutrient-rich media is indicated in orange and nutrient-poor media is indicated in blue) and a cell was kept mostly in one bypass channel. In every *measurement cycle*, the cell passes through the SMR for its buoyant mass to be measured and is briefly exposed to the opposing media. The zoom-in of buoyant mass versus time indicates the time of cell transit (black dots) along with the composition of media surrounding a cell. In order to keep the exposure short and consistent, the *measurement cycle* was programmed so that a cell spends more than 90% of its time in the current media (a) and less than 10% in the other media (b and c). At the end of the *media cycle* (indicated as the dotted line), the new *measurement cycle* began in the other side of bypass channel. The rate of media exchange surrounding a cell between two media cycles is dependent on the mixing of the new media and the old media, which leaks from the other side of bypass channel through the SMR at the moment of cell transit. Considering the 1-D lateral and longitudinal diffusion and geometry of the bypass channels and the SMR, it is conservatively estimated that, in the case of glucose, 90% of new media would surround the cell within 50 milliseconds after the transit, and more than 99% within 5 seconds.

**
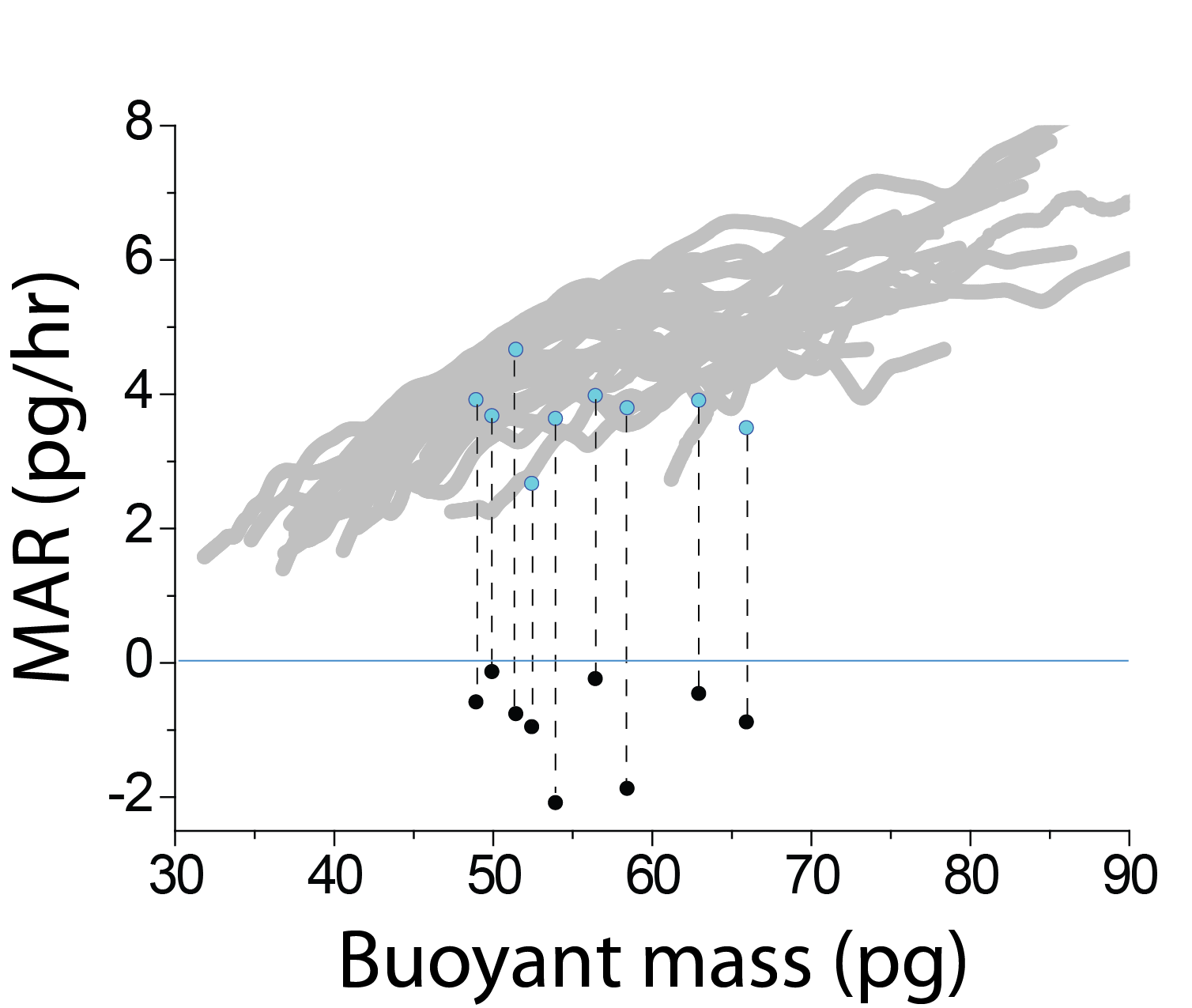
**

**Supplementary Figure 3. Nutrient input is required for mass accumulation.**

Growth of single FL5.12 cells is measured in normal media and in PBS for 30 minutes each. In contrast to the growth in normal media (blue dots), growth in PBS (black dots) is negative for all the cells measured. For reference, grey lines (obtained from *(1)*) show MARs that were continuously measured from 24 FL5.12 cells in normal media.

**
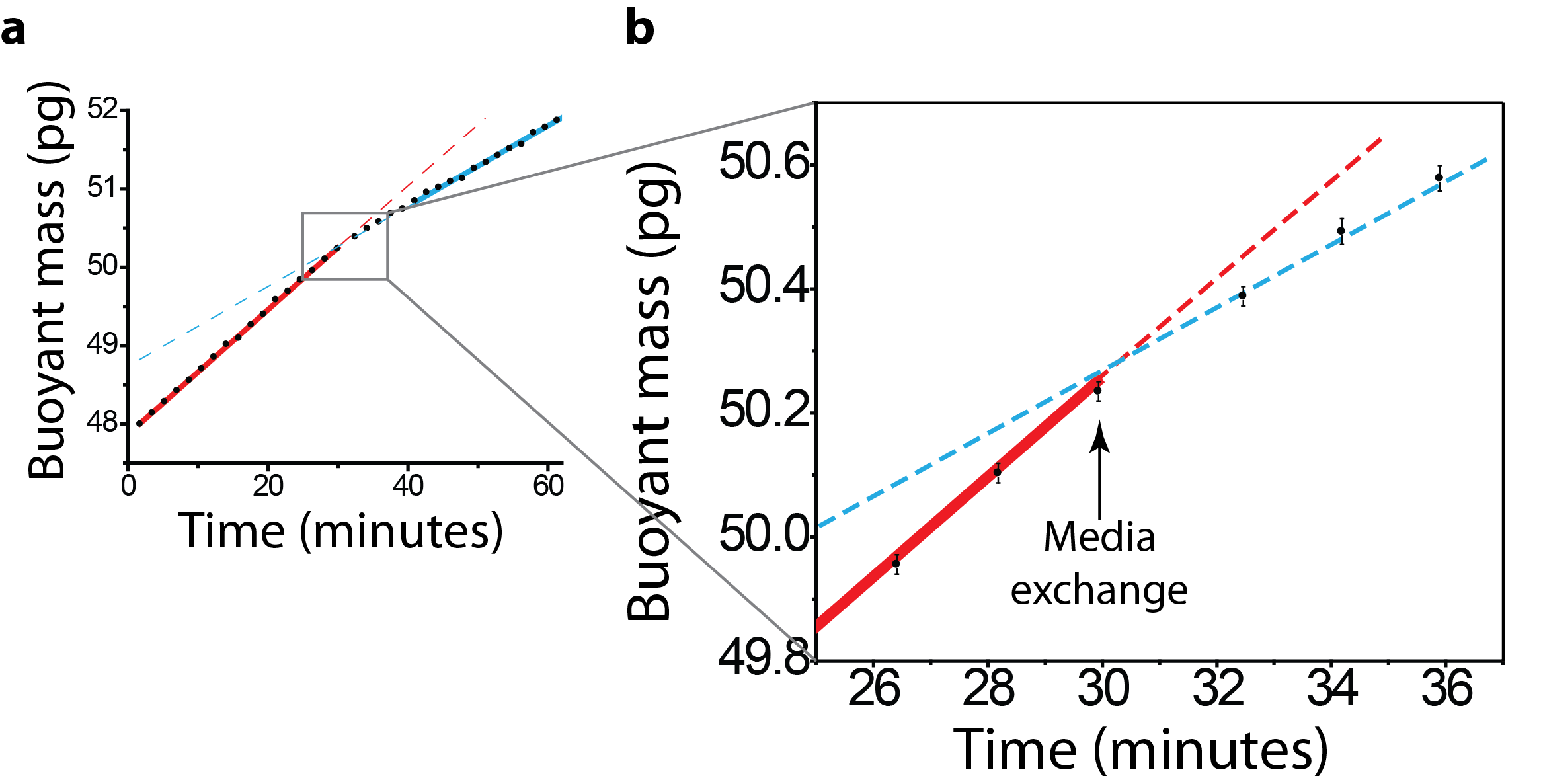
**

**Supplementary Figure 4. MAR changes within two minutes after nutrient depletion.**

(a) The plot shows representative MAR change when a FL5.12 cell is deprived of glutamine. In order to find the time of MAR change following nutrient depletion, we compared the linear lines extrapolated from the old (red line) and new (green line) MAR. Data points within the thick straight lines were used to determine the MAR. The thin dotted lines indicate the extrapolation of the MAR. By assuming that the growth rate can be transient for less than 10 minutes following depletion, we fit the buoyant mass from 10 to 30 minutes following depletion to determine the new MAR. (b) Individual buoyant mass measurements are shown with the error bars based on the root-mean-square (RMS) deviation from the linear fit. The first buoyant mass measured 2 minutes following the media exchange is significantly closer to the extrapolation of the new MAR.

**
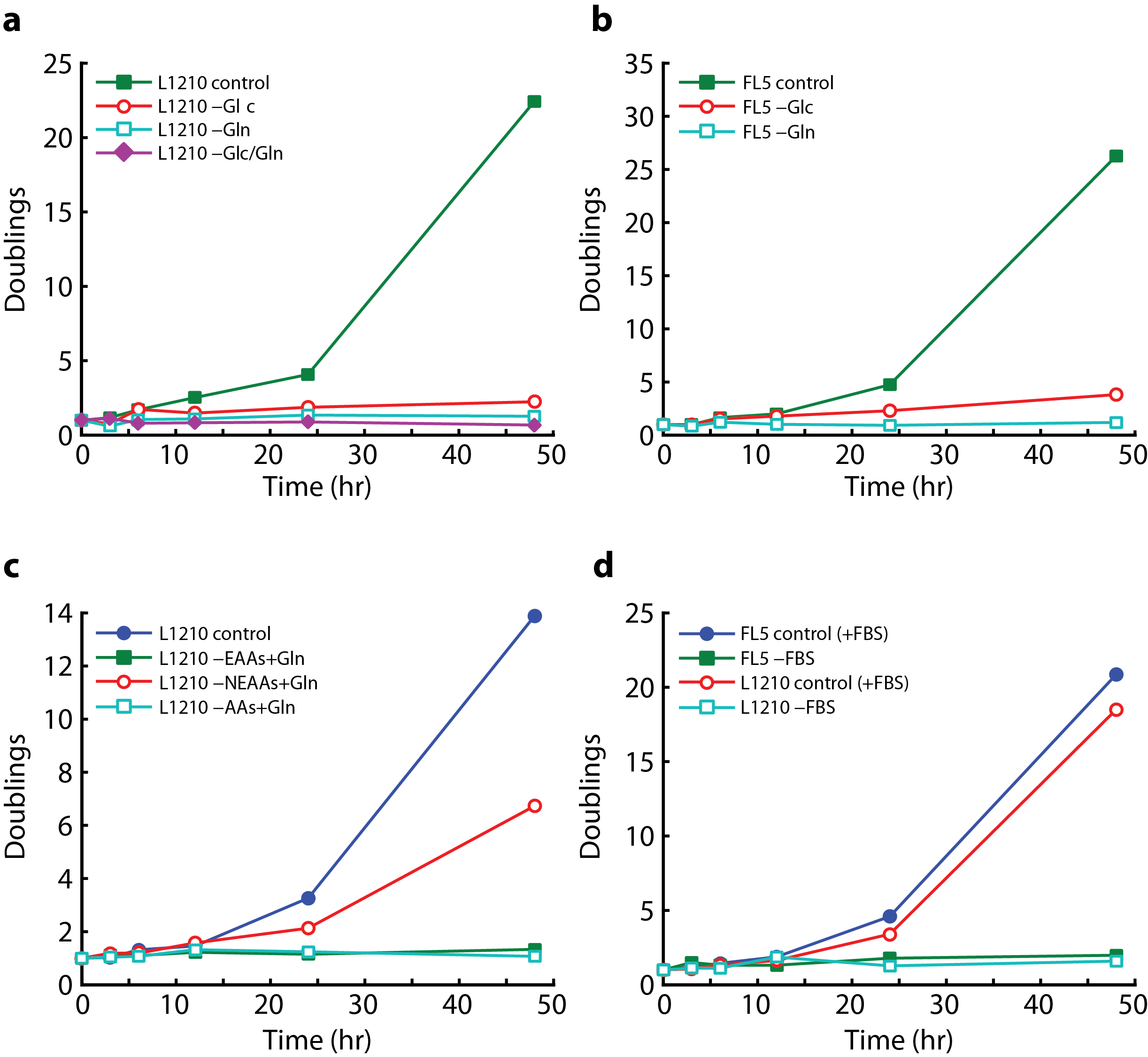
**

**Supplementary Figure 5. Long-term proliferation upon nutrient depletion.**

Bulk culture depletions of: (a) glucose (Glc) and glutamine (Gln) on L1210 cells, (b) Glc and Gln on FL5.12 cells, (c) essential amino acids (EAAs), non-essential amino acids (NEAAs), and all amino acids (AAs) on L1210 cells, (d) fetal bovine serum (FBS) in both L1210 and FL5.12. All amino acid depletions were performed with glutamine supplemented. **­­
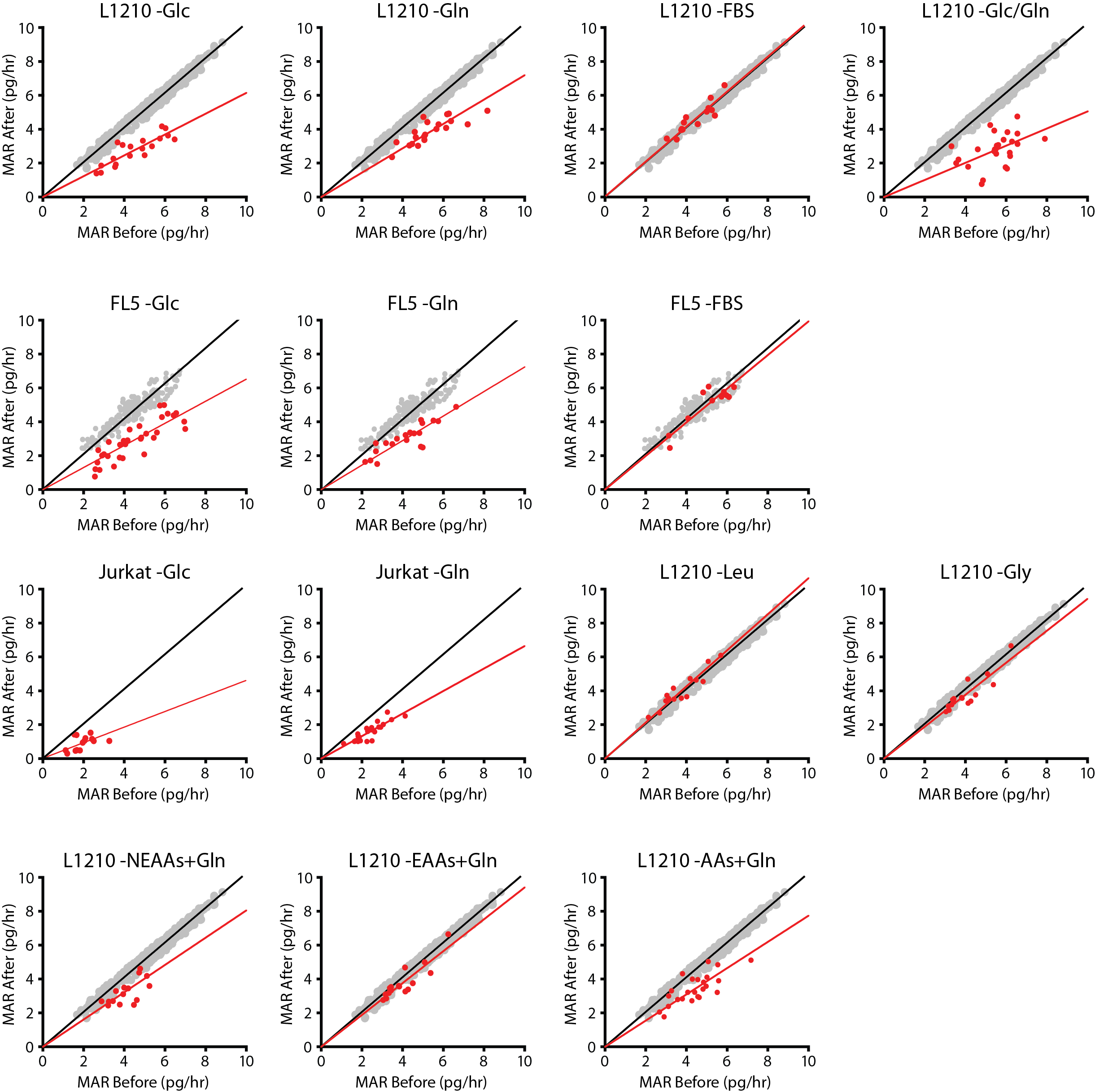
 ­­**

**Supplementary Figure 6. Single-cell MAR reduction in nutrient depletions.**

Grey dots and black lines indicate the control data and its linear fit. For Jurkat cells, black lines indicate a slope of 1, corresponding to no change of MAR Red dots and lines indicate the depletion data and its linear fit. Normalized slope±SE (as represented on Fig. 2 bar graph) and number of cells measured are: 0.600±0.021, n=20 (-Glc), 0.703±0.019, n=20 (-Gln), 1.015±0.023, n=14 (-FBS), 0.493±0.030, n=26 (-Glc/Gln), 0.627±0.022, n=32 (-Glc, FL5.12), 0.698±0.023, n=42 (-Gln, FL5.12), 0.955±0.031, n=10 (-FBS, FL5.12), 0.475±0.050, n=14 (-Glc, Jurkat), 0.665±0.023, n=23 (-Gln, Jurkat), 1.043±0.023, n=14 (-Leu), 0.903±0.027, n=19 (-Gly), 0.786±0.030, n=15 (-NEAAs+Gln), 0.919±0.027, n=18 (-EAAs+Gln), 0.755±0.026, n=30 (-AAs+Gln).

**
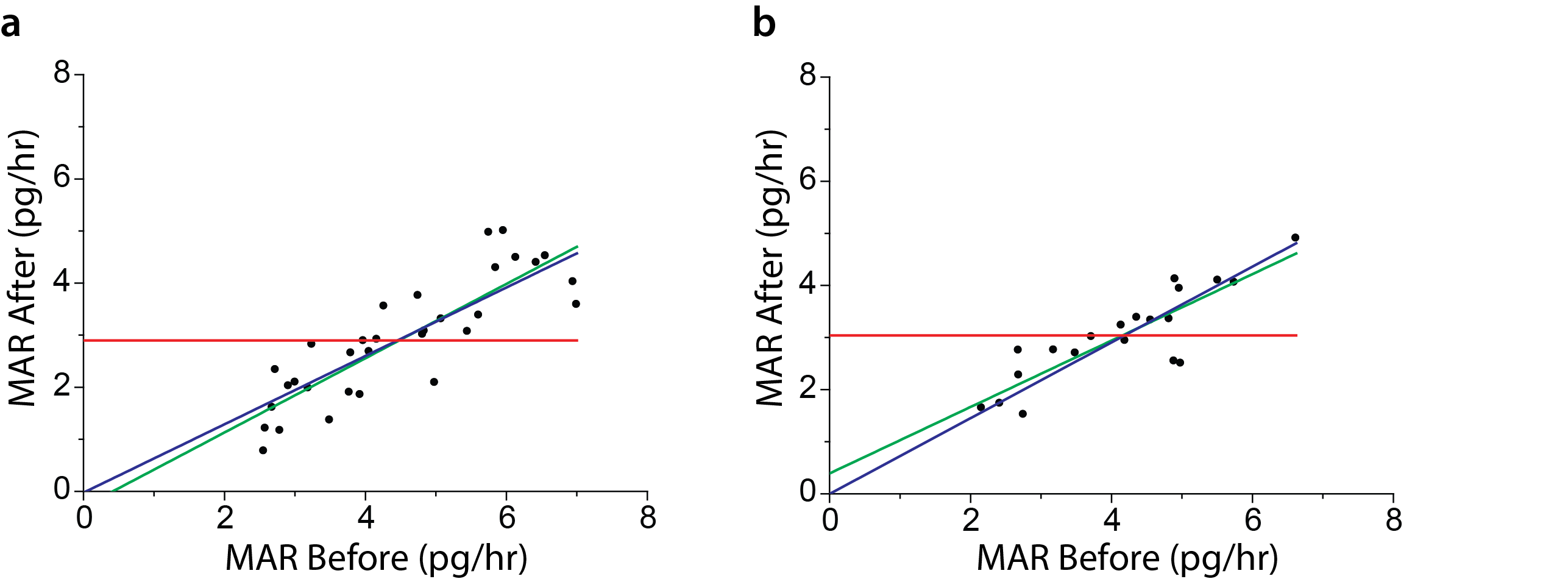
**

**Supplementary Figure 7. MAR reduction is proportional to the rate prior to depletion.**

Change of MAR of FL5.12 cells in the case of either glucose or glutamine depletion was fitted to three different linear lines: zero-intercept (blue), zero-slope (the red), or no constraints (green). (a) For glucose depletion, zero-intercept (N=32, slope=0.65±0.023, p<0.001, R^2^=0.96), zero-slope (slope=0, p=1, R^2^=0), and no constraints (slope=0.71±0.079, p<0.001, R^2^=0.72), respectively. (b) For glutamine depletion, zero-intercept (N=20, slope=0.73±0.024, p<0.001, R^2^=0.98), zero-slope (slope=0, p=1, R^2^=0), and no constraints (slope=0.63±0.087, p<0.001, R^2^=0.73), respectively**.**

**
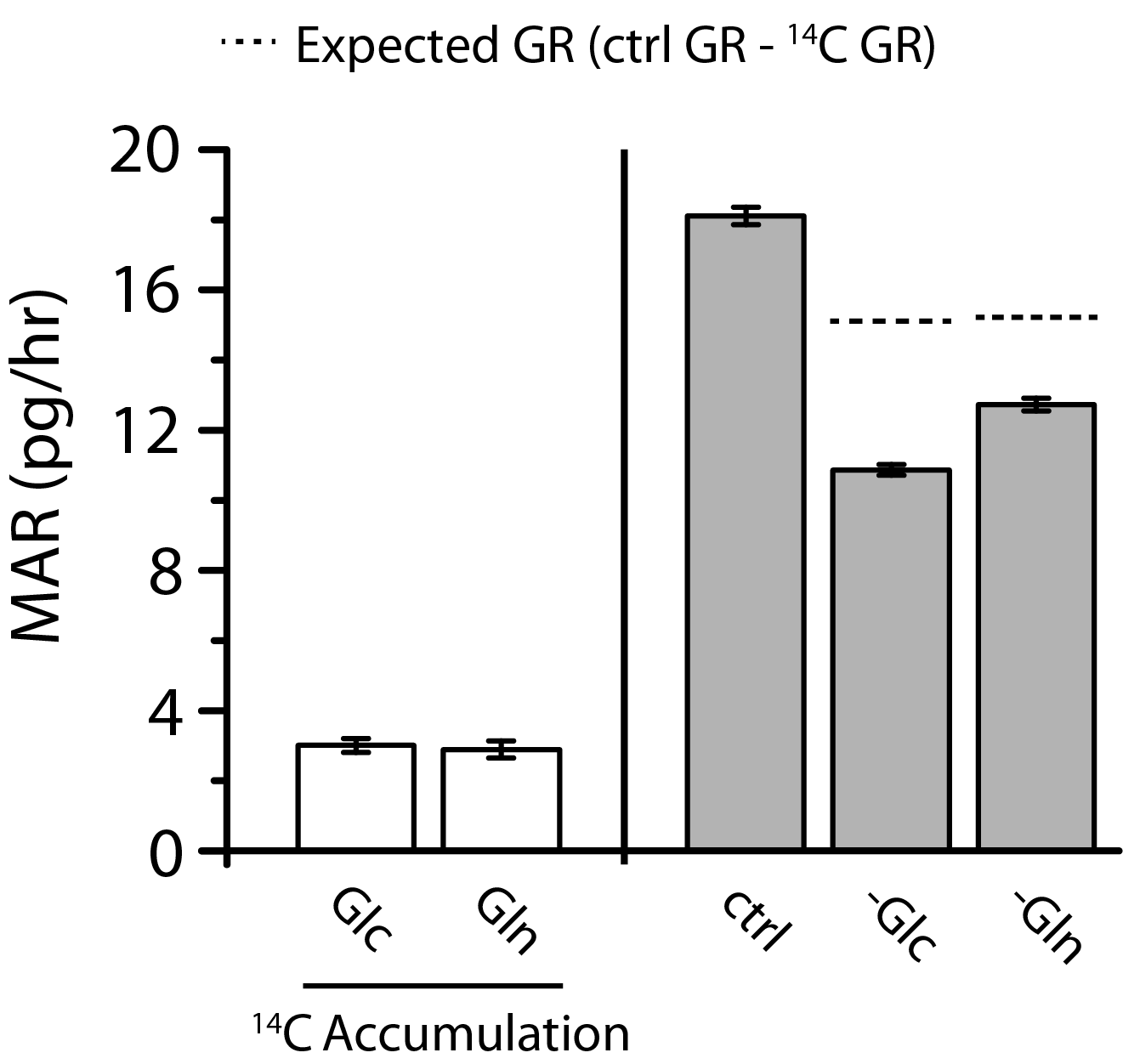
**

**Supplementary Figure 8. Glc/Gln mass accumulation rate is insufficient to account for lost MAR at depletion**

White bars denote upper limit of glucose and glutamine accumulation rates, derived from the measured per-cell ^14^C accumulation rates. Measured ^14^C values were converted to upper limits by multiplying by the ratio of total molecular mass to carbon mass, thus making the assumption that the entire mass of each glucose and glutamine molecule was incorporated into the cell. Grey bars represent the average MAR for control cells, and the expected average MAR for glucose and glutamine depletions derived by multiplying the growth rate of the control cells by the fraction that growth rate was reduced from the depletion.  This factor was determined by calculating the slope of the depletion responses shown in (Supplementary Fig. 6). Since measurements were in terms of buoyant mass, values were converted to dry mass by multiplying by a conversion factor derived from steady-state dry-density of L1210 cells and fluid density^3^. Dashed lines represent the expected average growth rate if the loss of molecular mass incorporation were the only contributor to the reduction of growth rate. Error bars represent standard error (SE).

**
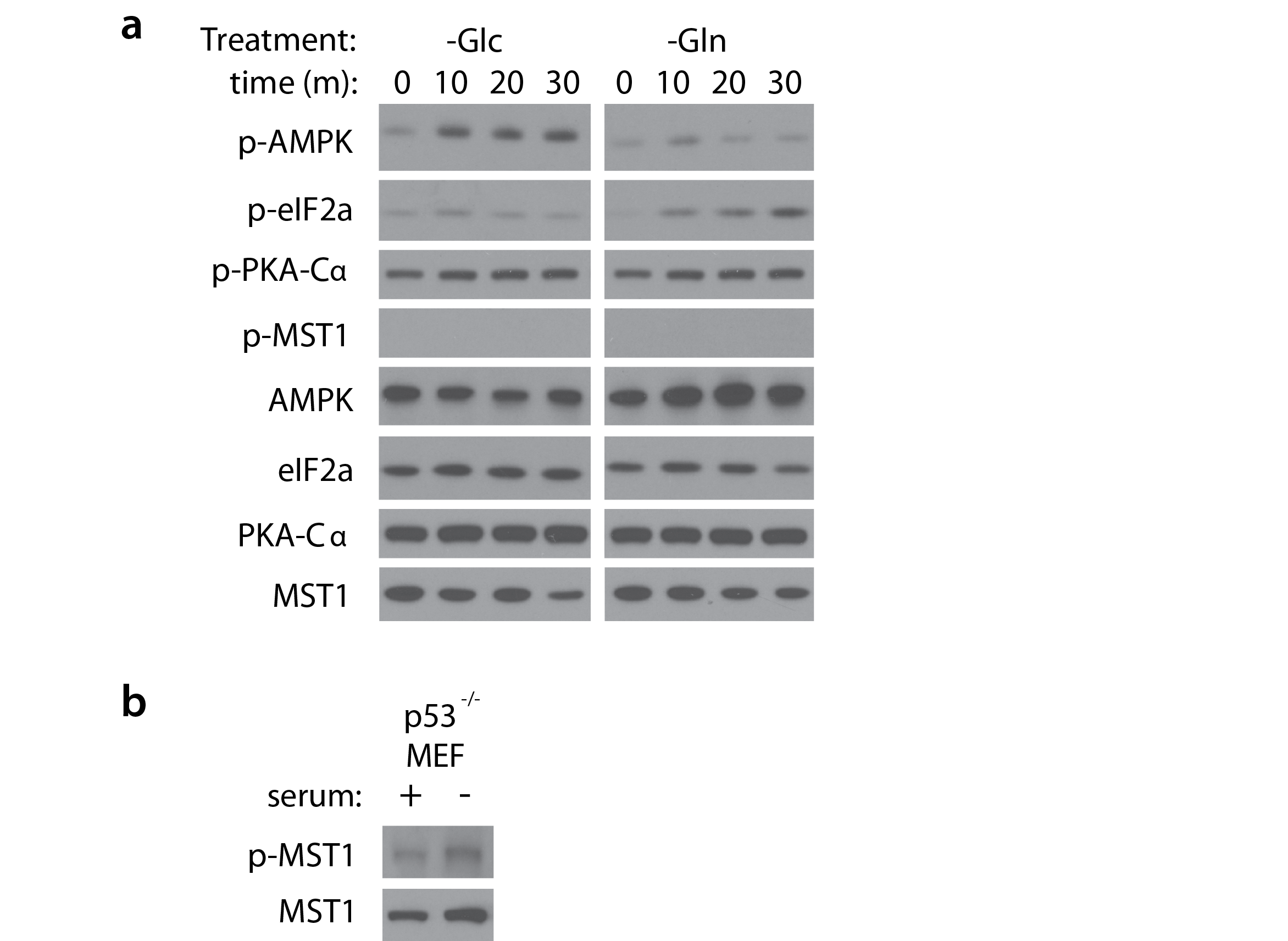
**

**Supplementary Figure 9. Known growth and nutrient sensing pathways are not involved in the instantaneous growth response in FL5.12 cell line**

(a) Immunoblot of phosphorylation of AMPK (Thr172), eiF2α (Ser51), PKA (Thr197), and MST1 (Thr183) in FL5.12 cells within 30 minutes after the depletion of glucose or glutamine. (b) Immunoblot of MST1 phosphorylation in mouse embryo fibroblasts (MEFs) depleted of serum as a positive antib­­ody control for murine cells.

**Supplementary Figure 10. Autophagy and apoptosis are not involved in instantaneous growth response**

Immunoblots of autophagy marker LC3 dimerization (a) and apoptosis marker caspase-3 cleavage (b) in response to depletion of glucose or glutamine over a 12 hour interval. Actin and ß-tubulin shown as loading controls. 250 nM Torin was used for LC3 positive control.

**
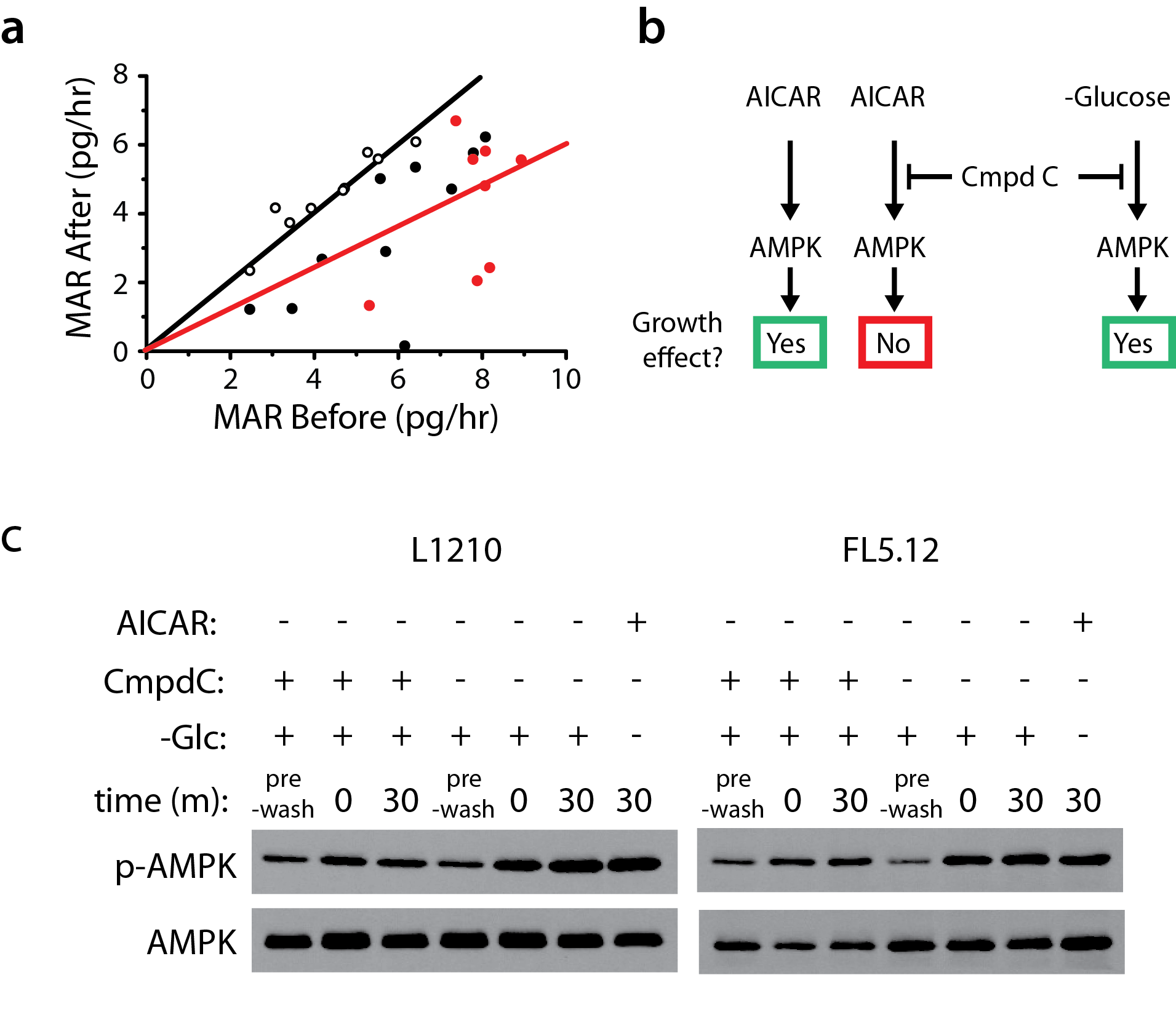
**

**Supplementary Figure 11. AMPK is not required for the growth response.**

(a) Black circles indicate the MAR change in the case of AICAR without Compound-C. White circles indicate AICAR with Compound-C. Red circles indicate glucose depletion with Compound-C. Black and red lines indicate the linear fit of control L1210 and L1210 -Glc, respectively. (b) Schematic of the results shown in panel a, and Figure 3c. AMPK is not necessary for the growth response upon glucose depletion. AICAR induces MAR reduction through phosphorylation of AMPK. Compound-C successfully inhibits AMPK activity as indicated by the abolishment of MAR reduction, but Compound-C does not abolish MAR reduction in the case of glucose depletion. (c) Immunoblot of AMPK and p-AMPK (Thr172) during glucose depletion, with and without Compound-C. AICAR positive control shown for comparison. 1mM AICAR and 5 µM Compound-C are used.

**
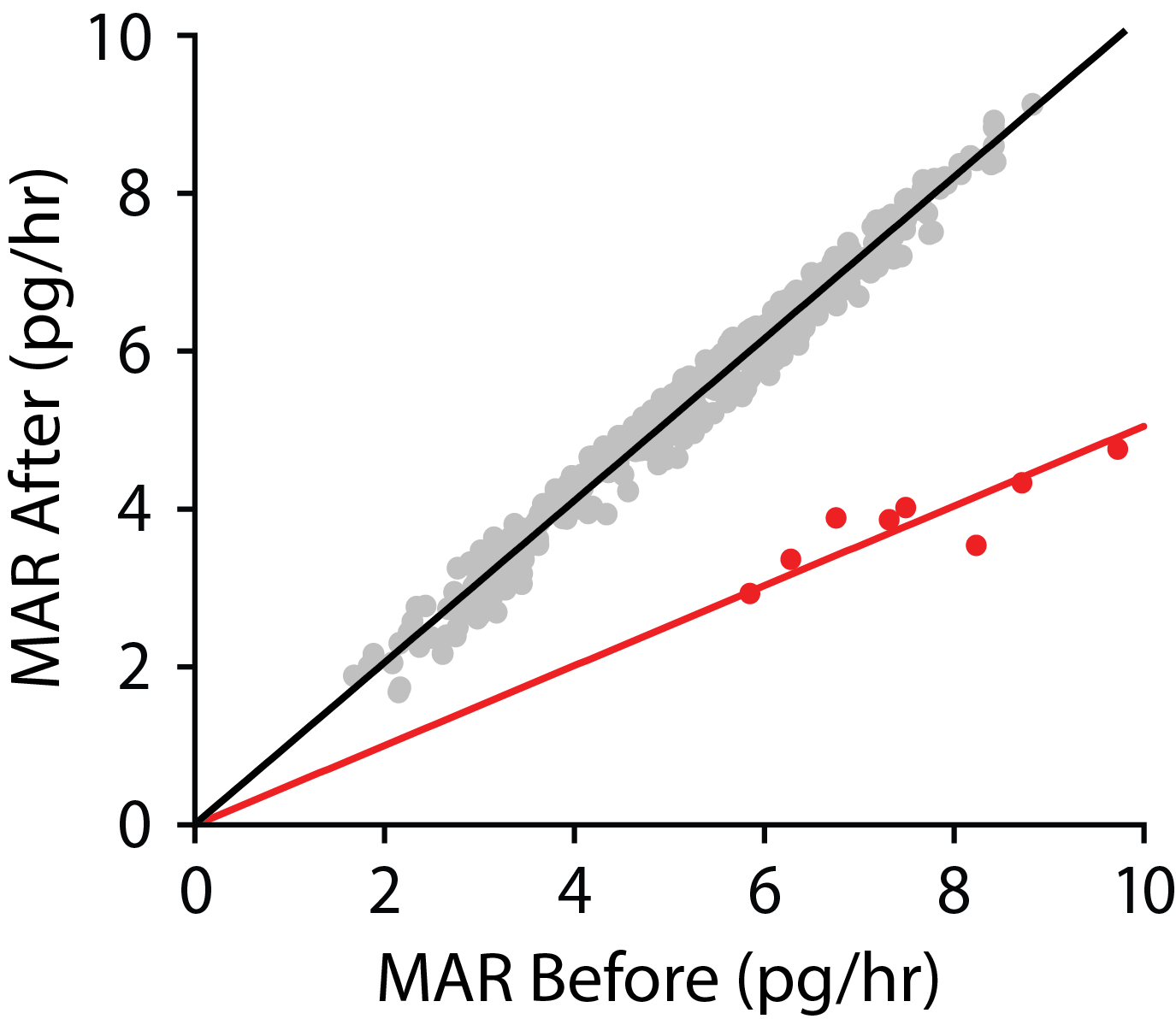
**

**Supplementary Figure 12. 2DG-supplementation does not rescue the MAR reduction following glucose depletion.**

Grey dots and black line indicate control data and its linear fit. Red dots and red line indicate data and linear fit for the depletion of glucose with 2-deoxyglucose (2DG) supplementation. Slope±SE and number of cells measured are 0.505±0.013 and n=9, respectively.

1. Son, S. *et al.* Direct observation of mammalian cell growth and size regulation. *Nat. Methods* **9,** 910–912 (2012).
2. Lee J., Bryan A.K., Manalis S.R., High precision particle mass sensing using microchannel resonators in the second vibration mode*. Rev. Sci. Instrum.* **82**, 023704 (2011).
3. Delgado F.F. *et al*. Intracellular water exchange for measuring the dry mass, water mass and changes in chemical composition of living cells. *PLos ONE* **8(9)** (2013).
